# Lung function and skin fibrosis changes as predictors of survival in SSc-associated interstitial lung disease: a EUSTAR study

**DOI:** 10.1093/rheumatology/keaf264

**Published:** 2025-06-03

**Authors:** Vincent Sobanski, Jeska de Vries-Bouwstra, Anna-Maria Hoffmann-Vold, Dörte Huscher, Margarida Alves, Marco Matucci-Cerinic, Gabriela Riemekasten, Mengtao Li, László Czirják, Otylia Kowal-Bielecka, Yannick Allanore, Nils Schoof, Oliver Distler, EUSTAR Collaborators, Silvia Bellando Randone, Ulrich Walker, Florenzo Iannone, Britta Maurer, Radim Becvar, Antonella Riccardi, Elise Siegert, Simona Rednic, Jérome Avouac, Carlomaurizio Montecucco, Patricia E Carreira, Cecilia Varju, Carlo Chizzolini, Andrea Doria, Bernard Coleiro, Armando Gabrielli, Dominique Farge Bancel, Paolo Airò, Alexandra Balbir-Gurman, Alessandro Giollo, Christopher Denton, Nemanja Damjanov, Jörg Henes, Vera Ortiz Santamaria, Stefan Heitmann, Bojana Stamenkovic, Carlo Francesco Selmi, Mohammed Tikly, Lidia P Ananieva, Ariane Herrick, Ulf Müller-Ladner, Merete Engelhart, Eric Hachulla, Valeria Riccieri, Ruxandra Maria Ionescu, Ana Maria Gheorghiu, Jörg Distler, Francesca Ingegnoli, Vanessa Smith, Francesco Paolo Cantatore, Susanne Ullman, Piotr Wiland, Marie Vanthuyne, Juan Jose Alegre-Sancho, Kristine Herrmann, Ellen De Langhe, Branimir Anic, Maria Üprus, Sule Yavuz, Carolina de, Souza Müller, Svetlana Agachi, D'Alessandro Mathieu, Kamal Solanki, Douglas Veale, Esthela Loyo, Edoardo Rosato, Edoardo Rosato, Cristina-Mihaela Tanaseanu, Rosario Foti, Codrina Ancuta, Peter Villiger, Jacob van Laar, Nihal Fathi, Paloma García de la Peña Lefebvre, Jean Sibilia, Ira Litinsky, Francesco Del Galdo, Lesley Ann Saketkoo, Eduardo Kerzberg, Washington Bianchi, Ivan Castellví, Doron Rimar, Maura Couto, François Spertini, Sarah Kahl, Vivien M Hsu, Thierry Martin, Lorinda S Chung, Tim Schmeiser, Dominik Majewski, Vera Bernardino, Konstantinos Fourlakis, Elena Rezus

**Affiliations:** Service de Médecine Interne, U1286 - INFINITE—Institute for Translational Research in Inflammation CHU Lille, Univ. Lille, Inserm, Lille, France; Institut Universitaire de France (IUF), Paris, France; Department of Rheumatology, Leiden University Medical Center, Leiden, The Netherlands; Department of Rheumatology, Oslo University Hospital, Oslo, Norway; Department of Rheumatology, University Hospital Zurich, University of Zurich, Zurich, Switzerland; Institute of Biometry and Clinical Epidemiology, and Berlin Institute of Health, Charité—Universitätsmedizin Berlin, Berlin, Germany; TA Inflammation Med, Boehringer Ingelheim International GmbH, Ingelheim am Rhein, Germany; Unit of Immunology, Rheumatology, Allergy and Rare Diseases (UnIRAR), and Inflammation, Fibrosis and Aging Initiative (INFLAGE), IRCCS San Raffaele Scientific Institute, Rome, Italy; Vita-Salute San Raffaele University, Milan, Italy; Department of Rheumatology and Clinical Immunology, University of Lübeck, Lübeck, Germany; Department of Rheumatology and Clinical Immunology, Peking Union Medical College Hospital, Chinese Academy of Medical Sciences and Peking Union Medical College, Beijing, China; Ministry of Education, Key Laboratory of Rheumatology and Clinical Immunology, Beijing, China; Department of Rheumatology and Immunology, Medical School, University of Pecs, Pecs, Hungary; Department of Rheumatology and Internal Medicine, Medical University of Bialystok, Bialystok, Poland; Rheumatology Department, Cochin Hospital APHP, INSERM U1016, Université Paris Cité, Paris, France; Integrated Evidence Generation TA WHC, Bayer AG, Berlin, Germany; Department of Rheumatology, University Hospital Zurich, University of Zurich, Zurich, Switzerland

**Keywords:** SSc-ILD, systemic sclerosis, interstitial lung disease, mortality, prognostic stratification

## Abstract

**Objectives:**

This study assessed how changes in lung function, skin fibrosis and digital ulceration (DU) burden predict mortality in patients with SSc-associated interstitial lung disease (SSc-ILD), the leading cause of death in SSc.

**Methods:**

Adult SSc-ILD patients from the European Scleroderma Trials and Research (EUSTAR) database enrolled since January 2009 with a date of diagnosis, a follow-up visit for change evaluation within 12 months plus a further visit or mortality information were eligible. Twelve-month changes in lung function (per cent predicted forced vital capacity [FVC%pred] and diffusing capacity of the lungs for carbon monoxide [DL_CO_%pred]), modified Rodnan skin score (mRSS) and change in DU burden were assessed for associations with survival, using multivariable Cox regression analyses adjusted for age, sex, smoking status and immunosuppressive therapy.

**Results:**

Of 893 SSc-ILD patients included, 94 (10.5%) died over a mean follow-up of 39.0 ± 23.9 months. Absolute deterioration in FVC >10%pred within 12 months (*n *= 78/638 evaluable) was predictive for decreased survival (hazard ratio [HR] 3.81; 95% CI 1.67–8.66), as were composite measures combining (i) >10% FVC decline or mRSS worsening (HR 2.82; 95% CI 1.43–5.56) and (ii) FVC decline ≥10% or 5–9% with DL_CO_ decline ≥15% (HR 3.42; 95% CI 1.68–7.00), but not changes in DL_CO_, mRSS or DU burden alone.

**Conclusions:**

Changes in lung function and skin fibrosis within 12 months should be considered when evaluating risk of mortality. The effect of pharmacological treatments aiming at stabilization of these variables should be evaluated prospectively in clinical trials.

Rheumatology key messages

- SSc progression, assessed by 12-month changes in lung function and skin fibrosis, can predict mortality.
- Regular assessments of lung function and mRSS are crucial for understanding SSc(-ILD) prognosis and management.
- Identification of SSc-ILD patients at higher risk of progression should be of priority for clinicians.

## Introduction

Interstitial lung disease (ILD) is a leading cause of morbidity and mortality in SSc, affecting >50% of patients [1], and is responsible for 15–33% of deaths in SSc [2–4]. Up to one-third of patients will develop progressive ILD within 12 months, leading to more extensive lung changes and worsening of fibrosis [1, 5]. Patients with ILD may develop loss of vital capacity during the first few years after SSc diagnosis [6], highlighting the need for early and regular screening [1]. Once ILD is identified, decisions regarding management can be challenging, given that some patients may not progress, and it may be difficult to identify progression at an early stage [7]. Age, male sex, history of smoking, low forced vital capacity (FVC), low diffusing capacity of the lungs for carbon monoxide (DL_CO_) and extent of lung fibrosis on high-resolution CT (HRCT) at baseline are associated with mortality in patients with SSc-associated ILD (SSc-ILD) [8–10].

Recently, agents such as pirfenidone, rituximab and tocilizumab have been investigated in clinical trials, alone or in combination with traditional immunosuppressants [11–13]. Patient selection for treatment is often based on the risk of adverse outcomes, including mortality. Thus it is important to be able to identify patients who are at highest risk of death [14].

Previous studies have suggested that short-term pulmonary function test (PFT) changes could be predictive of mortality, and composite indexes might add value [8, 15, 16]. In 162 patients of the Royal Brompton Hospital (London, UK) cohort, Goh *et al.* [17] determined that an FVC/DL_CO_ composite end point over a 12-month period (absolute FVC decline ≥10% predicted or 5–9% with absolute DL_CO_ decline ≥15% predicted) was predictive for mortality. In a *post hoc* analysis of Scleroderma Lung Studies (SLS) I and II [16], a decline in FVC and DL_CO_ over 2 years was a better predictor of mortality than baseline FVC and DL_CO_, with composite measures better predictors than changes in individual PFT scores. Changes in digital ulceration (DU) burden have also been associated with survival in a previous study [18]. Composite indices are increasingly used in clinical trials [19]. The current study therefore assessed the predictive ability of changes in lung function, skin fibrosis and DU burden, and combinations of these, for mortality in a large European cohort of patients with SSc-ILD. A plain language summary of this article can be found in Supplementary Data S1, available at *Rheumatology* online.

## Methods

### Cohort selection

**Figure 1.**
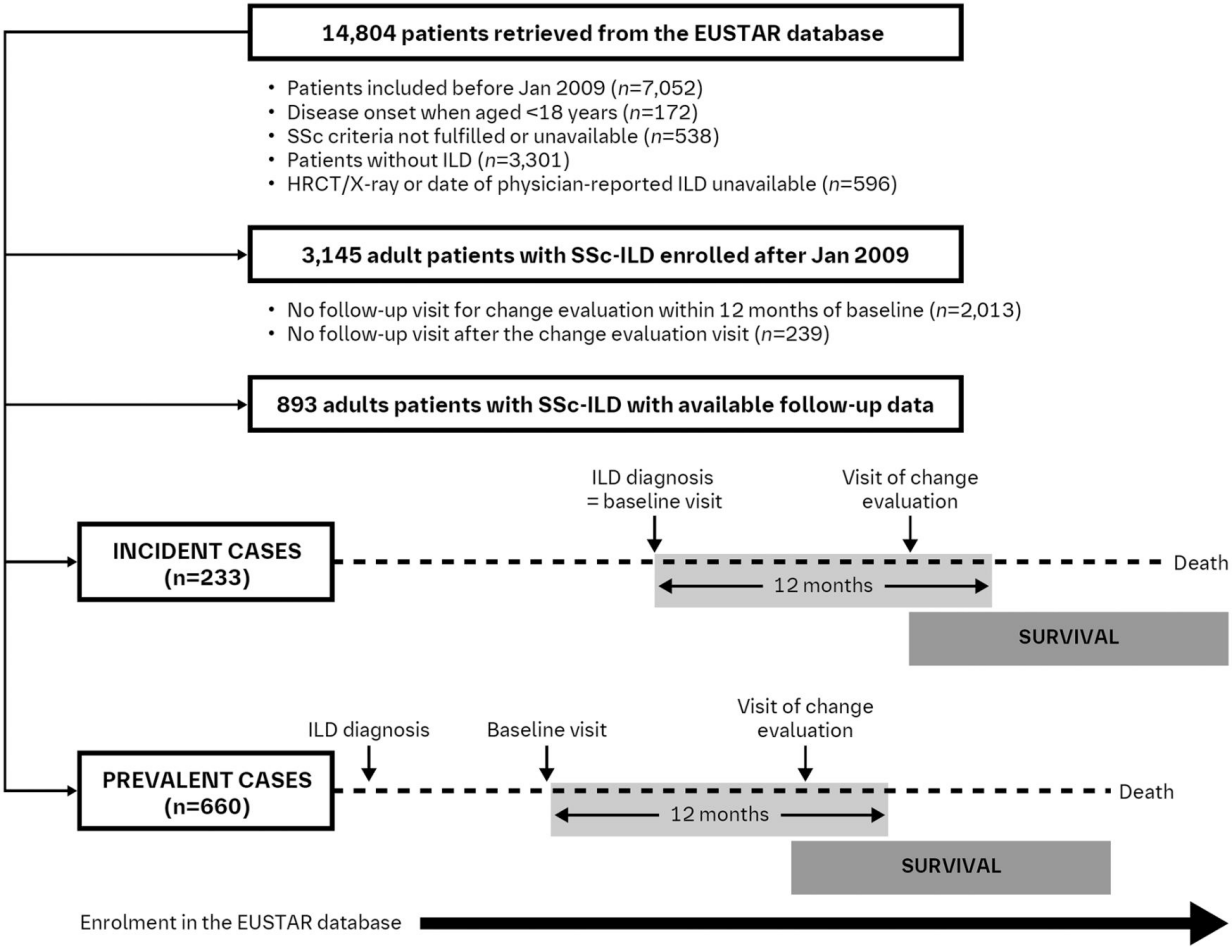
Flow chart of the patients through the study. EUSTAR, European Scleroderma Trials and Research; HRCT, high-resolution CT; ILD, interstitial lung disease; SSc-ILD, SSc-associated interstitial lung disease

This longitudinal cohort study was based on data from patients with SSc-ILD included in the European Scleroderma Trials and Research (EUSTAR) database [20], with visits recorded between 1 January 2009 and 3 May 2018. The eligibility criteria were: age ≥18 years; SSc according to the 1980 ACR or 2013 ACR/EULAR classification criteria [21, 22] and ILD according to HRCT and/or X-ray imaging and/or confirmed ILD diagnosis reported by the treating physician (Fig. 1).

The baseline visit was the date of enrolment into the database for prevalent cases (existing ILD for >6 months at enrolment into the database or unknown date of diagnosis) or the date of ILD diagnosis for incident cases (ILD diagnosis ≤6 months at inclusion or a new ILD diagnosis during follow-up). Follow-up was from the baseline visit until the last available documentation, withdrawal from the database or death. Patients were excluded if (i) they had no follow-up visit for change evaluation within 12 months after the baseline visit, or (ii) they had a follow-up visit for change evaluation after the baseline visit but no further visit and no information regarding mortality (Fig. 1). Pulmonary hypertension (PH), defined by right heart catheterization, could not be included because of missing data. The variable ‘PH suspected by echocardiogram’ was instead used, defined as increased systolic pulmonary arterial pressure or indirect signs of PH based on the clinician’s judgement. Treatment data were available for the selected cohort, but the indications for these therapies were unavailable. At the time of database extraction, data on pirfenidone or nintedanib were not registered.

### Outcomes

The cohort was analysed for associations with all-cause mortality of the following parameters (Table 1): (i) absolute changes from the baseline visit up to 12 months in per cent predicted values for FVC (FVC%pred) and DL_CO_ (DL_CO_%pred); (ii) absolute and relative changes from baseline to 12 months in modified Rodnan skin score (mRSS) and (iii) change in DU burden (development or persistence of DU vs remaining DU free and healed DU). Some parameters used in SSc clinical trials were not included due to rarity (history of scleroderma renal crisis ∼3%; not meaningful for multivariable analysis) or missing values (PH defined by right heart catheterization) in the EUSTAR cohort [23].

**Table 1.** Demographic and baseline characteristics of included and excluded SSc-ILD patients in the EUSTAR database

Variables	*n*	Included patients (*N* = 893)^a^	*n*	Excluded patients (*N* = 2252)^a^
Demographics, *n* (%)				
Age (years), mean ± S.D.	893	55.6 ± 12.8	2252	56.9 ± 13.4
Male sex	893	172 (19.3)	2252	422 (18.7)
Disease duration (years),^b^ median (IQR)	775	5.1 (2.5–10.4)	1939	5.6 (2.3–11.6)
Follow-up (months), mean ± S.D.	893	39.0 ± 23.9	1212	34.6 ± 25.7
Extra-pulmonary characteristics, *n* (%)				
dcSSc	849	370 (43.6)	2149	931 (43.3)
mRSS (units), mean ± S.D.	790	11.0 ± 9.2	2052	10.6 ± 9.1
2001 EUSTAR SSc activity score >3	892	201 (22.5)	2252	540 (24.0)
DU (past or active)	665	411 (61.8)	1574	971 (61.7)
Joint synovitis	874	133 (15.2)	2199	350 (15.9)
Muscle weakness	873	181 (20.7)	2193	473 (21.6)
Pulmonary hypertension suspected by echocardiography	726	142 (19.6)	1861	432 (23.2)
LVEF, mean ± S.D.	662	62.0 ± 6.3	1669	61.7 ± 7.2
Renal crisis	884	12 (1.4)	2236	53 (2.4)
ACA positive	811	139 (17.1)	1970	430 (21.8)
Anti-topo I positive	828	439 (53.0)	2035	1007 (49.5)
CRP elevation	831	252 (30.3)	2031	632 (31.1)
Immunosuppressive therapy^c^	893	469 (52.5)	2251	945 (42.0)
Lung-associated characteristics, *n* (%)				
Prevalent SSc-ILD	893	660 (73.9)	2252	1645 (73.0)
Incident SSc-ILD	893	233 (26.1)	2252	607 (26.0)
FVC (%pred), mean ± S.D.	754	86.4 ± 21.1	1843	85.9 ± 21.9
DL_CO_ (%pred), mean ± S.D.	708	60.6 ± 20.7	1729	60.7 ± 19.0
Dyspnoea NYHA ≥2	854	493 (57.7)	2072	1209 (58.3)

a Patients may not have had data for every variable, and results for each variable are expressed as a percentage of those with available data. For each group, *n* indicates the number of patients with available data.

b Calculated as difference between the date of the baseline visit and the date of the first non-RP symptom of the disease as reported by the patients.

c Defined as treatment with prednisone in doses >10 mg/day or any immunosuppressant (CYC, SSZ, MTX, LEF, AZA, MMF, ciclosporin A, D-Pen, rituximab, imatinib, anti-TNF-α, tocilizumab, abatacept or other biologic therapies) at baseline.

%pred, per cent predicted; DL_CO_, diffusing capacity of the lungs for carbon monoxide; DU, digital ulceration; EUSTAR, European Scleroderma Trials and Research; FVC, forced vital capacity; IQR, interquartile range; LVEF, left ventricular ejection fraction; mRSS, modified Rodnan skin score; NYHA, New York Heart Association; SSc-ILD, SSc-associated interstitial lung disease.

In addition, two composite disease progression definitions were analysed for association with survival: (i) lung progression defined as absolute decline in FVC ≥10%pred or absolute decline in FVC 5–9%pred with decline in DL_CO_ ≥15%pred [17], and (ii) lung or skin progression defined as absolute decline in FVC >10%pred or increase of mRSS >5 points and by >25% [24].

### Ethics

This study was conducted in accordance with the Declaration of Helsinki and was approved by the local ethical committees of the participating EUSTAR centres. All patients provided written informed consent for their data to be used for research purposes as required by the local ethics committees for this study.

### Statistical analyses

Comparisons were performed using Student’s *t* test or Mann–Whitney *U* test for continuous variables, and Fisher’s exact test or Chi-squared test for categorical variables.

Survival time was defined as the duration between the visit of change evaluation and the last visit with survival status, censored after 5 years of observation to maintain reasonable case numbers. The effect of changes in lung function, skin and DU burden on survival was evaluated using univariable analyses (Kaplan–Meier) for group comparisons and multivariable analyses (Cox proportional hazards models) adjusted for age, sex, smoking status and immunosuppressive therapy, as these parameters were judged to be clinically important for modifying major organ involvement. In multivariable analysis, the duration of ILD at enrolment as an additional confounder was considered for prevalent patients. For patients with an unknown date of ILD diagnosis, the duration of non-RP symptoms was used. Due to missing data on PH, as defined by right heart catheterization in the EUSTAR database, PH could not be included as a covariate in this analysis. Instead, PH suspected by echocardiogram was used as a covariate in a second multivariable analysis. Confounders with >20% missing values were omitted from multivariable analysis. *P*-values <0.05 were considered statistically significant. All analyses were conducted using IBM SPSS Statistics version 24.0 (IBM Corp, Armonk, NY, USA).

## Results

### Patient disposition

Of 14 804 patients screened, 3145 eligible patients with SSc-ILD were identified; 2013 had no follow-up visit within 12 months after the baseline visit (including 87 who died without a follow-up visit) and 239 had no follow-up after this evaluation. Therefore, the study population consisted of 893 patients, including 660 prevalent cases (73.9%) and 233 incident cases (26.1%), all of whom were included in the analysis (Fig. 1). Of these, 94 (10.5%) died over a mean follow-up of 39.0 ± 23.9 months. Of the 94 deaths, 81 (86.2%) were declared as SSc-related. Demographic and baseline characteristics of included and excluded patients are shown in Table 1. There were no significant differences between the study population and excluded patients with regard to sex, disease duration, proportion of dcSSc, joint and muscular involvement, CRP elevation and baseline PFT results. Differences were observed in other parameters, some of which may have been related to inclusion/exclusion criteria: patients in the excluded group were older, had a shorter follow-up, a higher prevalence of PH and ACA, and were receiving less immunosuppressive therapy than the study cohort. Detailed treatment data for patients are included in Supplementary Table S1, available at *Rheumatology* online.

### Changes during the first 12 months

The median time from baseline to the visit of change evaluation ranged between 8.5 and 9 months in groups according to the parameters in Table 2 and did not differ by >1 month between categories within each parameter group, with the exception of DU, for which there was a difference of 2 months.

**Table 2.** Disease parameters and categories of change assessed for prediction of mortality

Parameter	Categories	Patients meeting the endpoints
Absolute change from baseline in FVC	Increase or no decline in FVC	363 (56.9%)
	Decline in FVC >0–10%pred	197 (30.9%)
	Decline in FVC >10%pred	78 (12.2%)
Absolute change from baseline in DL_CO_	Increase or no decline in DL_CO_	318 (53.6%)
	Decline in DL_CO_ >0–10%pred	183 (30.9%)
	Decline in DL_CO_ >10–15%pred	39 (6.6%)
	Decline in DL_CO_ >15%pred	53 (8.9%)
Change from baseline in mRSS [25]	Improved or stable mRSS	509 (66.9%)
	Worsening in mRSS ≤5 points and/or ≤25%	183 (24.0%)
	Worsening in mRSS >5 points and >25%	69 (9.1%)
Change in DU burden	Remaining DU free or healed DU	612 (72.6%)
	Development or persistence of DU	231 (27.4%)
Lung progression: composite of FVC and DL_CO_ [17]	Increased/stable FVC or decline <10%pred, with decline in DL_CO_ <15%pred	539 (84.9%)
	Decline in FVC ≥10%pred or 5–9%pred, with decline in DL_CO_ ≥15%pred	96 (15.1%)
Lung and skin progression: composite of FVC and mRSS [24]	Increased/stable FVC or decline ≤10%pred, with improved/stable mRSS or worsening by ≤5 points or ≤25%	454 (76.7%)
	Decline in FVC >10%pred or worsening mRSS by >5 points and >25%	138 (23.3%)

%pred, per cent predicted; DL_CO_, diffusing capacity of the lungs for carbon monoxide; DU, digital ulceration; FVC, forced vital capacity; mRSS, modified Rodnan skin score.

At 12 months, 363 patients (56.9%) had no decline in FVC, 197 (30.9%) had a decline of >0–10%pred, and 78 (12.2%) had a decline in FVC >10%pred. The percentage of patients with changes in the disease parameters analysed at 12 months is shown in Table 2.

### Survival analyses

**Figure 2.**
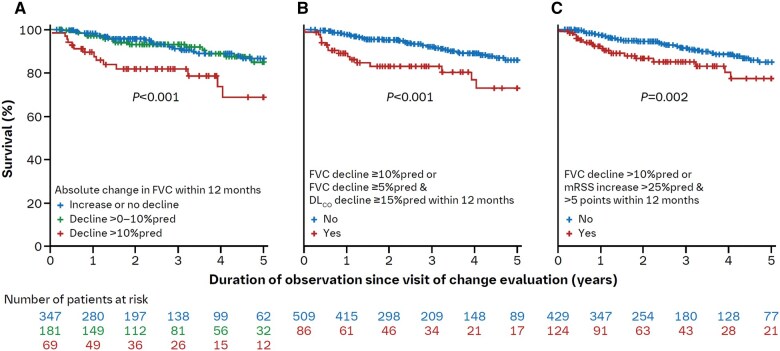
Overall survival according to (**A**) change in FVC, (**B**) change in FVC and/or DL_CO_ and (**C**) change in FVC and/or mRSS. All changes within 12 months of the baseline visit. Categories for FVC and DL_CO_ are based on %pred values. %pred, per cent predicted; DL_CO_, diffusing capacity of the lungs for carbon monoxide; FVC, forced vital capacity; mRSS, modified Rodnan skin score

In the whole cohort, 94 (10.5%) patients died during the follow-up period (mean 39.0 ± 23.9 months). Kaplan–Meier analyses identified significantly shorter survival in patients with progression of certain parameters within 12 months of the baseline visit: (i) those with a deterioration in FVC >10%pred (*P* < 0.001) (*n *= 78) (Fig. 2A); (ii) those with a decline in FVC ≥10%pred or a decline in FVC of 5–9%pred with a decline in DL_CO_ ≥15%pred (*n *= 96) (*P* < 0.001) (Fig. 2B) and (iii) those with a decline in FVC >10%pred or a worsening of mRSS >5 points and >25% (*P* = 0.002) (*n *= 138) (Fig. 2C). No associations with survival were found for isolated changes in DL_CO_%pred, mRSS or DU burden.

Multivariable Cox proportional hazard models with adjustment for age, sex, smoking status, duration of ILD and immunosuppressive therapy confirmed the findings of the Kaplan–Meier analyses, with hazard ratios (HRs) for mortality associated with changes in parameters as follows: (i) patients with a decline in FVC >10%pred (HR 3.81; 95% CI 1.67–8.66); (ii) patients with a decline in FVC ≥10%pred or a decline in FVC 5–9%pred with a decline in DL_CO_ ≥15%pred (HR 3.42; 95% CI 1.68–7.00) and (iii) patients with a decline in FVC >10%pred or an increase in mRSS >5 points and >25% (HR 2.82; 95% CI 1.43–5.56) (Table 3).

**Table 3.** Predictors of mortality in SSc-ILD in the EUSTAR database in uni- and multivariable Cox regression analyses

	Univariable analysis^a^		Multivariable analysis^a^	
	HR (95% CI)	*P*-value	HR (95% CI)	*P*-value
FVC % predicted change				
Increase or no decline	Reference		Reference	
Decline >0–10%	1.10 (0.58–2.10)	0.761	1.30 (0.60–2.84)	0.511
Decline >10%	**3.06 (1.59–5.88)**	**0.001**	**3.81 (1.67–8.66)**	**0.001**
DL_CO_ % predicted change				
Increase or no decline	Reference		Reference	
Decline >0–10%	0.94 (0.47–1.85)	0.847	0.66 (0.26–1.68)	0.386
Decline >10–15%	1.07 (0.33–3.55)	0.907	1.67 (0.48–5.80)	0.419
Decline >15%	0.96 (0.33–2.74)	0.934	0.33 (0.04–2.53)	0.283
mRSS change				
Decline or no increase	Reference		Reference	
Increase ≤5 points and/or ≤25%	0.68 (0.37–1.26)	0.219	0.93 (0.46–1.86)	0.827
Increase >5 points and >25%	1.17 (0.53–2.58)	0.706	2.06 (0.89–4.73)	0.090
DU				
DU free or healed DU	Reference		Reference	
New or persisting DU	0.97 (0.60–1.56)	0.888	0.75 (0.39–1.44)	0.392
Composite FVC and DL_CO_ change				
Increased/stable FVC or <10%, with DL_co_ decline <15%	Reference		Reference	
FVC decline ≥10% or 5–9%, with DL_CO_ decline ≥15%	**2.69 (1.50–4.83)**	**0.001**	**3.42 (1.68–7.00)**	**0.001**
Composite FVC and mRSS change				
FVC increased/stable/or decline ≤10%, with mRSS improved/stable or worsened by ≤5 points or ≤25%	Reference		Reference	
FVC decline >10%, or mRSS worsened by >5 points and >25%	**1.99 (1.13–3.52)**	**0.018**	**2.82 (1.43–5.56)**	**0.003**

a Models adjusted for age, sex, tobacco use and immunosuppressive therapy (both analyses) plus duration of ILD (multivariable analysis only).

DL_CO_, diffusing capacity of the lungs for carbon monoxide; DU, digital ulceration; EUSTAR, European Scleroderma Trials and Research; FVC, forced vital capacity; HR, hazard ratio; ILD, interstitial lung disease; mRSS, modified Rodnan Skin Score; SSc-ILD, SSc-associated interstitial lung disease. Bold text indicates significance: *P* < 0.05.

Because of missing data regarding PH defined by right heart catheterization in the EUSTAR database, this variable was not included in the analysis. However, a second multivariable analysis was performed as a sensitivity analysis, including the variable PH suspected by echocardiogram as a covariate. Results were similar although non-significant, with HRs for mortality associated with changes within 12 months of the baseline visit as follows: (i) HR 2.32 (95% CI 0.86–6.25) for decline in FVC >10%pred (*P* = 0.097); (ii) HR 2.08 (95% CI 0.86–5.06) for decline in FVC ≥10%pred or decline in FVC 5–9%pred with decline in DL_CO_ ≥15%pred (*P* = 0.105) and (iii) 2.30 (95% CI 0.97–5.47) for decline in FVC >10%pred or worsening in mRSS >5 points and >25% (*P* = 0.059) (Supplementary Table S2, available at *Rheumatology* online). When excluding patients with suspected PH, the HRs for mortality were as follows: (i) HR 2.34 (95% CI 0.72–7.56) for decline in FVC >10%pred (*P* = 0.155); (ii) HR 2.75 (95% CI 0.98–7.77) for decline in FVC ≥10%pred or decline in FVC 5–9%pred with decline in DL_CO_ ≥15%pred (*P* = 0.056) and (iii) 2.88 (95% CI 1.18–7.03) for decline in FVC >10%pred or worsening in mRSS >5 points and >25% (*P* = 0.020) (Supplementary Table S3, available at *Rheumatology* online).

## Discussion

These data indicate that changes in pulmonary function and skin fibrosis over 12 months were predictive for mortality in patients with SSc-ILD and should be considered in the prognostic stratification of these patients.

Given the high clinical heterogeneity of ILD in SSc, one of the main challenges that clinicians have to address when following SSc-ILD patients is to identify those at a higher risk of poor outcome [23]. PFTs offer a simple and non-invasive evaluation of lung involvement at initial diagnosis and during follow-up. However, PFTs do not discriminate between SSc patients with and without radiographic SSc-ILD [26]. Goh *et al.* developed an algorithm showing that extent of ILD >20% on HRCT, or lesser degrees of ILD combined with FVC <70%, might predict mortality [9]. This has been replicated in a subsequent study [27]. Until recently, baseline FVC has been an unadjusted predictor of mortality in multiple studies, but was not identified as an independent predictor in any multivariable analyses [8]. DL_CO_ has been identified in multiple studies as a significant predictor of mortality in both unadjusted and adjusted analyses, probably because DL_CO_ is a marker of ILD and PH, two major contributors to mortality [8]. The widespread use of FVC may underestimate the potential for other predictors of SSc-ILD progression. Failing to fully identify treatment effects in SSc-ILD randomized controlled trials, which was described in a recent systematic review, highlights the need to pursue efforts to identify the best sole or composite PFT surrogate marker for SSc-ILD [28].

FVC is one of the most reproducible physiological assessments of lung disease, although definitions of progression used in idiopathic pulmonary fibrosis (IPF) and SSc trials, such as a  ≥ 10% relative decline in FVC, may not reflect the natural history of decline in SSc-ILD [29]. A recent study found that the FVC in SLS I and II, measured at specialist centres in the USA, had acceptable test–retest reliability and that small changes in FVC (3–5.3%) correlated with patient-reported outcomes and findings on HRCT, defining these as minimal clinically important differences [30]. Following a consensus exercise in 2015, the Outcome Measures in Rheumatology Connective Tissue Disease (CTD)-associated ILD working group proposed a clinically meaningful outcome in clinical trials of CTD-associated ILD (≥10% relative decline in FVC or a ≥ 5% to <10% relative decline in FVC with ≥15% relative decline in DL_CO_) [31]. Data-driven approaches to validate these results in different cohorts and randomized controlled trials are needed. In this study, an absolute decline in FVC ≥10% or 5–10% with a decline in DL_CO_ ≥15% within 12 months of the baseline visit was associated with mortality, independent of age, sex, smoking status and immunosuppressive therapy. This is consistent with a recent study by Goh *et al.* that examined the prognostic significance of PFTs at 1 and 2 years against 15-year survival in patients with SSc-ILD, which found a composite measure to be the best predictor for mortality [17]. One-year PFT changes were predictive only in patients with extensive lung disease. In those with less fibrosis, composite indices combining smaller declines in FVC (<10%) with DL_CO_ decline (≥15%) at 2 years were more predictive for mortality. The extent of lung fibrosis on HRCT has only been recorded in the EUSTAR database since 2013, and therefore this item could not be evaluated. A recent *post hoc* analysis of SLS I and II also showed that decline in FVC and DL_CO_ over 2 years was a better predictor of mortality than baseline FVC and DL_CO_, with composite measures better predictors than changes in individual PFT scores [16]. Together, these results suggest that short-term variations in surrogate measures of SSc-ILD progression might have important effects on long-term outcomes [16].

The main caveats to using FVC and/or DL_CO_ decline to define SSc-ILD worsening are that the reported time to decline is highly dependent on the intervals between PFTs (likely to be influenced by disease severity) and that FVC or DL_CO_ may vary during follow-up, so that patients fulfilling the criteria of ‘significant decline’ at one visit may not fulfil them at the following visit. An analysis of SSc-ILD progression in the EUSTAR database showed a highly variable and heterogeneous disease course [32]. Due to a lack of standardization, the formula for calculating FVC%pred and DL_CO_%pred may also vary between centres. Using a linear mixed model that integrates all serial PFTs in SSc-ILD patients, Le Gouellec *et al.* found that during follow-up of 75 patients, FVC was stable while DL_CO_ significantly decreased (–1.5 ± 0.3%/year) [33]. Presence or history of DU and presence of PH at baseline or during follow-up were associated with a faster decline of DL_CO_ over time. Some studies have suggested that FVC decline is most rapid earlier in the course of SSc-ILD and that ILD might be more stable after the first 4 years following ILD diagnosis [34, 35]. However, one study has suggested that this impression could be an artefact secondary to survival bias. Indeed, a plateau in the progression of FVC was apparent in the full cohort analysis but disappeared with stratification into prognostic subgroups to account for survival bias. Patients had distinct patterns of progression that remained relatively consistent during long-term follow-up. They further showed that recent change in FVC could not predict future change in FVC within shorter follow-up intervals; however, patients with a decline in DL_CO_ tended to have a continuous decrease in the subsequent year [15]. A EUSTAR study, which focused on long-term progression patterns in patients with SSc-ILD by assessing FVC decline through the 12 ± 3-month trial and in the 5-year follow-up period, showed that FVC had heterogeneous and variable trajectories. The proportion of patients with SSc-ILD who experienced FVC decline of ≥5% during the initial 12 ± 3-month period was 27%, and in each 12-month period over the mean 5-year follow-up, 23–27% of patients experienced progression [32].

The high heterogeneity in SSc does not allow a single treatment approach to be identified and also affects enrolment of patients in clinical trials [27]. European evidence-based consensus statements have provided several ways of assessing progression (in treated or untreated patients): changes in PFTs (FVC and DL_CO_ absolute values or FVC decline); changes in extent of fibrosis or pattern on HRCT; changes in exercise-induced oxygen desaturation and worsening of clinical symptoms [36]. Several predictive models using demographic and clinical data have been developed to assess prognosis in patients with ILD. The GAP (sex, age and lung physiology [i.e. FVC and DL_CO_]) model was originally developed to assess 1-, 3- and 5-year mortality in IPF but has been applied and validated in patients with CTD, including SSc [37]. The SADL (smoking history, age, DL_CO_) model has been validated specifically in SSc patients to predict all-cause mortality in SSc-ILD [10]. The SPAR (SpO_2_, arthritis) model utilizes optimal cut-offs for desaturation of oxygen with 6-min walk testing in combination with the presence of arthritis, which were both identified as independent predictors for ILD progression in patients with mild SSc-ILD [38]. In the current study, although change in mRSS alone was not associated with survival, unlike in a recent EUSTAR study in unselected patients with dcSSc [39], the composite of FVC decline 5–9% or mRSS change predicted mortality, but with a lower HR than FVC decline >10%. An explanation could be that progressive skin fibrosis, as assessed by mRSS, has been associated with lung function decline, assessed by changes in FVC, and worse survival during follow-up [39].

Immunosuppressive therapies remain the mainstay of treatment for SSc-ILD [40], but the drug armamentarium is still relatively limited for this severe and life-threatening complication of SSc. Numerous targeted therapies are being evaluated [41]. Studies with nintedanib (a tyrosine kinase inhibitor), tocilizumab (an IL-6 receptor inhibitor) and rituximab (an anti-CD20 antibody) have shown alleviation of the annual rate of decline in FVC among patients with SSc-ILD [11, 12, 42]. Nintedanib is approved for patients with SSc-ILD worldwide, tocilizumab is approved for patients with SSc-ILD in the United States and rituximab is approved for patients with SSc in Japan [43]. Riociguat, a soluble guanylate cyclase stimulator that has been evaluated in patients with early dcSSc (RISE-SSc), has shown some potential efficacy signals in this population [44, 45]. Additionally, many drugs, either approved or in development for SSc and progressive pulmonary fibrosis (including SSc-ILD), target additional, different mechanisms [13, 46, 47]. For example, pirfenidone, an antifibrotic agent that is approved for use in IPF and evaluated in guidelines for progressive pulmonary fibrosis, has been assessed in patients with SSc-ILD [13], with a recent American Thoracic Society guideline recommendation for additional research into its efficacy and safety in this population, either alone or in combination with MMF [40]. The phosphodiesterase-4 inhibitor nerandomilast has shown antifibrotic and anti-inflammatory properties in preclinical studies [46] and stabilized lung function over 12 weeks in patients with IPF [47]. Nerandomilast is currently being investigated, along with amlitelimab, in a Phase IIb trial over 52 weeks compared with placebo in patients with SSc-ILD (CONQUEST, NCT06195072) [44]. The B-cell-targeted mAb belimumab, currently approved for the treatment of LN and SLE, is being evaluated in adults with SSc-ILD (NCT05878717) [48], while the anti-type 1 IFN receptor antibody anifrolumab is being evaluated in a randomized Phase III trial in SSc (DAISY, NCT05925803). Patients are being stratified based on the presence or absence of ILD at baseline, with changes from baseline in FVC in patients with SSc-ILD as a key secondary end point [49].

Overall, this analysis showed that changes in lung function and skin fibrosis are predictive of mortality in SSc-ILD. Therefore, stabilizing these parameters might be the first step in a treat-to-target strategy before approaches leading to improvement become available. These findings are important as they demonstrate the link between short-term changes and mortality, independent of treatment, and will be applicable as new treatments are introduced.

The main strengths are the number of participants and the use of both clinical and physiological variables. Limitations include the *post hoc* nature of the analyses, precluding standardization in the selection, initial evaluation and follow-up visits of patients. Indeed, several patients had no follow-up visit within 12 months after the baseline visit or after the 12 months’ evaluation, as expected for an observational registry study. Also, all patients had radiologically confirmed ILD, but not all had HRCT data available, and the date of confirmed ILD diagnosis was missing for 47% of patients with prevalent ILD. In these patients, the onset of non-RP symptoms was used as an indicator of disease progression. This likely had minimal impact on the results, as half of the patients with a recorded ILD onset date had a confirmed ILD diagnosis for over 3 years. Additionally, previous EUSTAR data indicate that approximately one-quarter of patients experience progressive ILD, defined as an FVC decline of ≥5%, in each 12-month period over a 5-year follow-up, suggesting a relatively stable progression rate [32]. Finally, exhaustive haemodynamic data are not available for large cohorts as right heart catheterization is usually performed only in case of suspected PH. In the multivariable sensitivity analysis, which included the variable PH suspected by echocardiogram in the model, results were similar for the different predictors, although not statistically significant. This can be explained by a lower number of cases included in the analysis (726 patients out of 893 had available data) or collinearity between variables (DL_CO_ and presence of PH or ILD, for example). When patients with suspected PH were excluded, HRs for mortality were stable.

These data confirm that a decline in FVC or a combined FVC/DL_CO_ or FVC/mRSS deterioration are predictive for mortality and may be particularly valuable for interpreting clinical trials in SSc-ILD. These data give insights into how changes in these parameters may affect mortality. These findings also support the regular assessment of decline in FVC%pred, which should be taken into consideration when evaluating risk of mortality.

## Supplementary Material

keaf264_Supplementary_Data

## Data Availability

Data are available upon reasonable request.
