# Supplementary material for: Lung function and skin fibrosis changes as predictors of survival in SSc-associated interstitial lung disease: a EUSTAR study
Source: Rheumatology (Oxford). 2025 Jun 3;64(10):5344–53. doi: 10.1093/rheumatology/keaf264 (PMC12494203; doi:10.1093/rheumatology/keaf264)
Supplement: keaf264_Supplementary_Data [file keaf264_supplementary_data.zip › keaf264_Supplementary_Data/EUSTAR SSc mortality manuscript_supplement_13March2025 clean.docx]

**Supplementary Table S1.** Treatment data for patients from the EUSTAR database included in the current analysis

| **Variables** | ***n*** | **Included patients**  **(*N*=893)^a^** | ***n*** | **Excluded patients**  **(*N*=2,252)^a^** |
| --- | --- | --- | --- | --- |
| Immunosuppressive therapy,^b^ *n* (%) | 893 | 469 (52.5) | 2,251 | 945 (42.0) |
| CS >10 mg/d | 892 | 63 (6.3) | 2,250 | 135 (7.1) |
| CYC | 893 | 99 (9.1) | 2,249 | 186 (11.1) |
| SSZ | 893 | 5 (0.3) | 2,248 | 4 (0.6) |
| MTX | 892 | 125 (12.5) | 2,247 | 267 (14.0) |
| LEF | 892 | 6 (0.7) | 2,246 | 15 (0.7) |
| AZA | 892 | 92 (8.9) | 2,249 | 188 (10.3) |
| Mycophenolic acid | 893 | 166 (15.3) | 2,248 | 315 (18.6) |
| Cyclosporine A | 893 | 6 (0.9) | 2,247 | 21 (0.7) |
| D-Pen | 893 | 3 (0.4) | 2,246 | 9 (0.3) |
| At least one biologic therapy administered | 893 | 34 (3.8) | 2,248 | 111 (4.9) |
| Rituximab | 893 | 22 (3.0) | 2,248 | 73 (2.5) |
| TNF-α antagonist | 893 | 3 (0.2) | 2,248 | 2 (0.3) |
| Tocilizumab | 893 | 4 (1.0) | 2,248 | 28 (0.4) |
| Abatacept | 893 | 3 (0.2) | 2,248 | 2 (0.3) |
| Other biologic therapy | 893 | 2 (0.4) | 2,247 | 11 (0.2) |
| ERA | 893 | 155 (17.4) | 2,250 | 277 (12.3) |
| PDE-5i | 892 | 83 (9.3) | 2,249 | 163 (7.2) |
| Prostacyclins | 893 | 191 (21.4) | 2,252 | 361 (16.0) |

^a^Some patients were on multiple biologic therapies. ^b^Excluding chloroquine/HCQ and glucocorticoids <=10mg/d; ERA: endothelin receptor antagonists; EUSTAR, European Scleroderma Trials and Research; PDE-5i: phosphodiesterase-5 inhibitors.

**Supplementary Table S2.** Predictors of mortality in SSc-ILD in the EUSTAR database, with pulmonary hypertension suspected by echocardiogram included in the multivariable analysis

|  | **Univariable analysis** | | **Multivariable analysis^a^** | |
| --- | --- | --- | --- | --- |
|  | **HR (95% CI)** | ***P*-value** | **HR (95% CI)** | ***P*-value** |
| **FVC%pred change**  Increase or no decline  Decline >0–10%  Decline >10% | Reference  1.10 (0.58–2.10)  **3.06 (1.59–5.88)** | 0.761  **0.001** | Reference  1.04 (0.44–2.49)  2.32 (0.86–6.25) | 0.929  0.097 |
| **DL_CO_%pred change**  Increase or no decline  Decline >0–10%  Decline >10–15%  Decline >15% | Reference  0.94 (0.47–1.85)  1.07 (0.33–3.55)  0.96 (0.33–2.74) | 0.847  0.907  0.934 | Reference  0.37 (0.10–1.30)  1.24 (0.28–5.48)  0.44 (0.06–3.40) | 0.366  0.775  0.428 |
| **mRSS change**  Decline or no increase  Increase ≤5 points and/or ≤25%  Increase >5 points and >25% | Reference  0.68 (0.37–1.26)  1.17 (0.53–2.58) | 0.219  0.706 | Reference  0.86 (0.36–2.05)  2.31 (0.84–6.32) | 0.735  0.103 |
| **Digital ulceration**  No digital ulceration  Occurrence of digital ulceration | Reference  0.97 (0.60–1.56) | 0.888 | Reference  0.82 (0.39–1.70) | 0.587 |
| **Composite FVC and DL_CO_ change**  Increased/stable FVC or <10%, with DL_CO_ decline <15%  FVC decline ≥10% or 5–9%, with DL_CO_ decline ≥15% | Reference  **2.69 (1.50–4.83)** | **0.001** | Reference  2.08 (0.86–5.06) | 0.105 |
| **Composite FVC and mRSS change**  FVC increased/stable or decline ≤10%, with mRSS improved/stable or worsened by ≤5 points or ≤25%  FVC decline >10%, or mRSS worsened by >5 points and >25% | Reference  **1.99 (1.13–3.52)** | **0.018** | Reference  2.30 (0.97–5.47) | 0.059 |

^a^Models adjusted for age, sex, tobacco use, pulmonary hypertension suspected by echocardiogram and immunosuppressive therapy.

%pred, per cent predicted; DLco, diffusing capacity of the lungs for carbon monoxide; EUSTAR, European Scleroderma Trials and Research; FVC, forced vital capacity; HR, hazard ratio; mRSS, modified Rodnan skin score; SSc-ILD, SSc-associated interstitial lung disease.

**Supplementary Table S3.** Predictors of mortality in SSc-ILD in the EUSTAR database, excluding cases with pulmonary hypertension suspected by echocardiogram

|  | **Univariable analysis** | | **Multivariable analysis^a^** | |
| --- | --- | --- | --- | --- |
|  | **HR (95% CI)** | ***P*-value** | **HR (95% CI)** | ***P*-value** |
| **FVC%pred change**  Increase or no decline  Decline >0–10%  Decline >10% | Reference  1.07 (0.45–2.56)  1.76 (0.58–5.35) | 0.873  0.319 | Reference  0.82 (0.31–2.22)  2.34 (0.72–7.56) | 0.704  0.155 |
| **DL_CO_%pred change**  Increase or no decline  Decline >0–10%  Decline >10–15%  Decline >15% | Reference  0.55 (0.18–1.65)  1.42 (0.33–6.23)  0.91 (0.21–3.99) | 0.286  0.639  0.904 | Reference  0.65 (0.20–2.04)  2.21 (0.46–10.60)  0.73 (0.09–5.76) | 0.456  0.321  0.761 |
| **mRSS change**  Decline or no increase  Increase ≤5 points and/or ≤25%  Increase >5 points and >25% | Reference  0.85 (0.36–2.00)  1.52 (0.52–4.42) | 0.714  0.444 | Reference  0.94 (0.37–2.43)  2.66 (0.87–8.09) | 0.901  0.086 |
| **Digital ulceration**  No digital ulceration  Occurrence of digital ulceration | Reference  1.15 (0.60–2.20) | 0.683 | Reference  0.66 (0.26–1.65) | 0.371 |
| **Composite FVC and DL_CO_ change**  Increased/stable FVC or <10%, with DL_CO_ decline <15%  FVC decline ≥10% or 5–9%, with DL_CO_ decline ≥15% | Reference  1.82 (0.69–4.83) | 0.230 | Reference  2.75 (0.98–7.77) | 0.056 |
| **Composite FVC and mRSS change**  FVC increased/stable or decline ≤10%, with mRSS improved/stable or worsened by ≤5 points or ≤25%  FVC decline >10%, or mRSS worsened by >5 points and >25% | Reference  1.78 (0.77–4.08) | 0.177 | Reference  **2.88 (1.18–7.03)** | **0.020** |

^a^Models adjusted for age, sex, tobacco use and immunosuppressive therapy.

%pred, per cent predicted; DLco, diffusing capacity of the lungs for carbon monoxide; EUSTAR, European Scleroderma Trials and Research; FVC, forced vital capacity; HR, hazard ratio; mRSS, modified Rodnan skin score; SSc-ILD, SSc-associated interstitial lung disease.
