# Supplementary material for: Lung function and skin fibrosis changes as predictors of survival in SSc-associated interstitial lung disease: a EUSTAR study
Source: Rheumatology (Oxford). 2025 Jun 3;64(10):5344–53. doi: 10.1093/rheumatology/keaf264 (PMC12494203; doi:10.1093/rheumatology/keaf264)
Supplement: keaf264_Supplementary_Data [file keaf264_supplementary_data.zip › keaf264_Supplementary_Data/rhe-24-2887-File002.docx]

Supplementary Data S1

Plain language summary

Systemic sclerosis (SSc) is a rare autoimmune disease where the person’s immune (defence) system attacks healthy tissue in their body. SSc causes inflammation and scarring (fibrosis) of the skin and other tissues throughout the body. It also affects the vessels in the fingers and toes causing painful sores, called digital ulcers. Fibrosis in the lungs can lead to a serious condition called interstitial lung disease (ILD). ILD presents with cough, difficulty in breathing, and trouble moving around. People with SSc and ILD (SSc-ILD) may have a reduced life span compared with people who have SSc alone.

Our study included 893 people with SSc-ILD from the European Scleroderma Trials and Research (EUSTAR) database. We wanted to know if worsening of their lungs, skin thickening, and digital ulcers over the 12 months following their enrolment in the database were related to survival.

We also looked at survival over a 3-year period. During this time, 94 people died. Survival was shorter in people whose lung function worsened compared with people whose lung function did not worsen; people who had either worsening lung function or worsening skin thickening had shorter survival. Worsening of skin thickening or digital ulcers alone did not seem to affect survival.

This research suggests that monitoring changes in lung function and skin fibrosis for short periods (for example, a year) could help doctors predict which people with SSc-ILD are at highest risk of dying from their disease.

Word count: 237/250

Supplementary Data S2

EUSTAR collaborators

(1) Silvia Bellando Randone, Department of Medicine, Section of Rheumatology, University of Florence, Florence, Italy; (2) Ulrich Walker, Department of Rheumatology, University Hospital Basel, Basel, Switzerland; (4) Florenzo Iannone, Rheumatology Unit-DiMIMP, School of Medicine, University of Bari, Bari, Italy; (6) Britta Maurer, Department of Rheumatology, University Hospital Zürich, Zürich, Switzerland; (7) Radim Becvar, Institute of Rheumatology, 1st Medical School, Charles University, Prague, Czech Republic; (13) Antonella Riccardi, Department of Clinical and Experimental Medicine ‘F-Magrassi’ II Policlinico, Unit of Rheumatology, Naples, Italy; (15) Elise Siegert, Department of Rheumatology, Charité University Hospital, Berlin, Germany; (16) Simona Rednic, Department of Rheumatology, University of Medicine and Pharmacy ‘Iuliu Hatieganu’, Cluj-Napoca, Romania; (17) Jérome Avouac, Department of Rheumatology, University Paris Descartes and Cochin Hospital, Paris, France; (19) Carlomaurizio Montecucco, Unita’Operativa e Cattedra di Reumatologia, IRCCS Policlinico San Matteo, Pavia, Italy; (23) Patricia E. Carreira, Division of Rheumatology, Hospital 12 de Octubre, Madrid, Spain; (25) Cecilia Varju, Department of Immunology and Rheumatology, Faculty of Medicine, University of Pecs, Pecs, Hungary; (28) Carlo Chizzolini, Department of Immunology and Allergy, University Hospital, Geneva, Switzerland; (31) Andrea Doria, Rheumatology Unit, Department of Clinical and Experimental Medicine, University of Padova, Padova, Italy; (33) Bernard Coleiro, ‘Stella Maris’, Balzan, Malta; (34) Armando Gabrielli, Dipartimento di Scienze Cliniche e Molecolari, Clinica Medica, Universita Politecnica delle Marche, Ancona, Italy; (35) Dominique Farge Bancel, Department of Internal Medicine, Hospital Saint-Louis, Paris, France; (38) Paolo Airò, Spedali Civili di Brescia, Servizio di Reumatologia, Allergologia e Immunologia Clinica, Brescia, Italy; (42) Alexandra Balbir-Gurman, B. Shine Rheumatology Unit, Rambam Health Care Campus, Rappaport Faculty of Medicine, Technion, Haifa, Israel; (50) Alessandro Giollo, Unita di Reumatologia, AOUI, Verona, Italy; (52) Christopher Denton, Centre for Rheumatology, Royal Free and University College London Medical School, Royal Free Campus, London, United Kingdom; (55) Nemanja Damjanov, Institute of Rheumatology, Belgrade, Serbia; (56) Jörg Henes, Medizinische Universitätsklinik, Abt. II (Onkologie, Hämatologie, Rheumatologie, Immunologie, Pneumologie), Tubingen, Germany; (57) Vera Ortiz Santamaria, Rheumatology Granollers General Hospital, Barcelona, Spain; (58) Stefan Heitmann, Department of Rheumatology, Marienhospital Stuttgart, Stuttgart, Germany; (73) Bojana Stamenkovic, Institute for Prevention, Treatment and Rehabilitation of Rheumatic and Cardiovascular Diseases, Niska Banja, Serbia; (74) Carlo Francesco Selmi, Division of Rheumatology and Clinical Immunology Humanitas Clinical and Research Center, BIOMETRA Department, University of Milan, Milan, Italy; (77) Mohammed Tikly, Rheumatology Unit, Department of Medicine, Chris Hani Baragwanath Hospital and University of the Witwatersrand, Johannesburg, South Africa; (78) Lidia P. Ananieva, Institute of Rheumatology, Russian Academy of Medical Science, Moscow, Russia; (80) Ariane Herrick, Hope/Hospital University of Manchester Rheumatic Diseases Centre, Clinical Sciences, Salford, United Kingdom; (81) Ulf Müller-Ladner, Justus Liebig University Giessen, Department of Rheumatology and Clinical Immunology, Kerckhoff-Klinik, Bad Nauheim, Germany; (86) Merete Engelhart, Department of Rheumatology, University Hospital of Gentofte, Hellerup, Denmark; (93) Eric Hachulla, Department of Internal Medicine, L'Hôpital Claude Huriez, Lille, France; (94) Valeria Riccieri, Department of Internal Medicine and Medical Specialties, ‘Sapienza’ University of Rome, Rome, Italy; (96) Ruxandra Maria Ionescu, Department of Rheumatology, St. Mary Hospital, Carol Davila, University of Medicine and Pharmacy, Bucharest, Romania; (100) Ana Maria Gheorghiu, Department of Internal Medicine and Rheumatology, Cantacuzino Hospital, Carol Davila University of Medicine and Pharmacy, Bucharest, Romania; (106) Jörg Distler, Department of Internal Medicine 3, University Hospital Erlangen, Erlangen, Germany; (110) Francesca Ingegnoli, Division of Rheumatology, Istituto Gaetano Pini, Department of Clinical Sciences and Community Health, University of Milan, Milan, Italy; (113) Vanessa Smith, University of Ghent, Department of Rheumatology, Ghent, Belgium; (115) Francesco Paolo Cantatore, U.O. Reumatologia-Università degli Studi di Foggia, Ospedale ‘Col. D’Avanzo’, Foggia, Italy; (116) Susanne Ullman, University Hospital of Copenhagen, Department of Dermatology D-40, HS-Bispebjerg Hospital, Copenhagen, Denmark; (120) Piotr Wiland, Department of Rheumatology and Internal Diseases, Wroclaw University of Medicine, Wroclaw, Poland; (122) Marie Vanthuyne, Universite catholique de Louvain, Brussels, Belgium; (123) Juan Jose Alegre-Sancho, Hospital Universitario Dr Peset, Valencia, Spain; (125) Kristine Herrmann, Division of Rheumatology, Department of Medicine III/Department of Dermatology, University Medical Center Carl Gustav Carus, Technical University of Dresden, Dresden, Germany; (126) Ellen De Langhe, Catholic University of Leuven, Department of Rheumatology, Leuven, Belgium; (128) Branimir Anic, Marko Baresic, Miroslav Mayer, University Hospital Centre Zagreb, Division of Clinical Immunology and Rheumatology, Department of Medicine, Zagreb, Croatia; (130) Maria Üprus, Kati Otsa, East-Tallinn Central Hospital, Department of Rheumatology, Tallinn, Estonia; (133) Sule Yavuz, University of Marmara, Department of Rheumatology, Istanbul, Turkey; (135) Carolina de Souza Müller, Hospital de Clínicas da Universidade Federal do Paraná, Curitiba, Brazil; (137) Svetlana Agachi, Municipal Centres of Research in Scleroderma, Hospital ‘Sacred Trinity’, Department of Rheumatology/Department of Rheumatology, Republican Clinical Hospital, Chisinau, Republic of Moldova; (142) D'Alessandro Mathieu, Alessandra Vacca, University of Cagliari-Policlinico, Monserrato, Italy; (148) Kamal Solanki, Waikato Hospital, Rheumatology Unit, Hamilton City, New Zealand; (149) Douglas Veale, Department of Rheumatology, Bone and Joint Unit, St. Vincent’s University Hospital, Dublin, Republic of Ireland; (152) Esthela Loyo, Carmen Tineo, Reumatologia e Inmunologia Clinica, Hospital Regional Universitario José Maria Cabral y Bàez, Clinica Corominas, Santiago, Dominican Republic; (158) Edoardo Rosato, Centro per la Sclerosi Sistemica-Dipartimento di Medicina Clinica, Universita La Sapienza, Policlinico Umberto I, Rome, Italy; (158) Edoardo Rosato, Ege University, Faculty of Medicine, Department of Internal Medicine, Division of Rheumatology, Izmir, Turkey; (160) Cristina-Mihaela Tanaseanu, Clinical Emergency Hospital St. Panteleimon, Bucharest, Romania; (161) Rosario Foti, U.O. di Reumatologia, A.O.U. Policlinico Vittorio Emanuele, Catania, Italy; (162) Codrina Ancuta, Division of Rheumatology and Rehabilitation, Center for Biomedical Research, European Center for Translational Research, “GR.T.Popa” University of Medicine and Pharmacy, Rehabilitation Hospital, Iasi, Romania; (164) Peter Villiger, Sabine Adler, Department of Rheumatology and Clinical Immunology/Allergology, Inselspital, University of Bern, Switzerland; (165) Jacob van Laar, James Cook University, Middlesbrough, United Kingdom; (168) Nihal Fathi, Assiut and Sohag University Hospital, Rheumatology Department, Assiut, Egypt; (169) Paloma García de la Peña Lefebvre, Jorge Juan Gonzalez Martin, Hospital Universitario Madrid Norte Sanchinarro, Madrid, Spain; (172) Jean Sibilia, Hôpital de Hautepierre – Hôpitaux Universitaires de Strasbourg, Service de Rhumatologie, Strasbourg Cedex, France; (173) Ira Litinsky, Department of Rheumatology, Tel Aviv Sourasky Medical Center, Tel Aviv, Israel; (175) Francesco Del Galdo, Scleroderma Programme, Institute of Molecular Medicine, Division of Musculoskeletal Diseases, University of Leeds, Leeds, United Kingdom; (177) Lesley Ann Saketkoo, Tulane University Lung Center, Tulane/University Medical Center Scleroderma and Sarcoidosis Patient Care and Research Center, New Orleans, USA; (178) Eduardo Kerzberg, Osteoarticular Diseases and Osteoporosis Centre, Pharmacology and Clinical Pharmacological Research Centre, School of Medicine, University of Buenos Aires, Rheumatology and Collagenopathies Department, Ramos Mejia Hospital, Buenos Aires, Argentina; (179) Washington Bianchi, Breno Valdetaro Bianchi, Department of Rheumatology, Santa Casa da Misericórdia do Rio de Janeiro, Rio de Janeiro, Brazil; (180) Ivan Castellví, Hospital de la Santa Creu i Sant Pau, Barcelona, Spain; (183) Doron Rimar, Rheumatology Unit, Bnai Zion Medical Center, Haifa, Israel; (184) Maura Couto, Unidade de Reumatologia de Viseu, Centro Hospitalar Tondela-Viseu (Unidade de Reumatologia), Viseu, Portugal; (185) François Spertini, Department of Rheumatology, Clinical Immunology and Allergy, Centre Hospitalier Universitaire Vaudois, Lausanne, Switzerland; (187) Sarah Kahl, Universitätsklinikum Schleswig-Holstein, Campus Lübeck, Innere Medizin/Rheumatologie/Immunologie, Rheumaklinik Bad Bramstedt, Bad Bramstedt, Germany; (188) Vivien M. Hsu, UMDNJ – Scleroderma Program, Clinical Research Center – Robert Wood Johnson Medical School, New Brunswick, USA; (189) Thierry Martin, Clinical Immunology and Internal Medicine, National Referral Center for Systemic Autoimmune Diseases, Strasbourg, France; (191) Lorinda S. Chung, Department of Dermatology, Stanford University School of Medicine, Stanford, USA; (192) Tim Schmeiser, Krankenhaus St. Josef, Wuppertal-Elberfeld, Germany; (193) Dominik Majewski, Department of Rheumatology and Internal Medicine, University of Poznan, Poznan, Poland; (198) Vera Bernardino, Unidade de Doencas Auto-Imunes, Hospital Curry Cabral, Centro Hospitalar Lisboa Central, Lisbon, Portugal; (199) Konstantinos Fourlakis, Universitätsklinik Lübeck, Lübeck, Germany; (202) Elena Rezus, Division of Rheumatology & Rehabilitation, “GR.T.Popa” University of Medicine and Pharmacy, Rehabilitation Hospital, Iasi, Romania.
